# Linking neuronal lineage and wiring specificity

**DOI:** 10.1186/s13064-018-0102-0

**Published:** 2018-04-13

**Authors:** Hongjie Li, S. Andrew Shuster, Jiefu Li, Liqun Luo

**Affiliations:** 1 Department of Biology and Howard Hughes Medical Institute, Stanford University, Stanford, CA 94305, USA; 2 Neurosciences Graduate Program, Stanford University, Stanford, CA 94305 USA

## Abstract

Brain function requires precise neural circuit assembly during development. Establishing a functional circuit involves multiple coordinated steps ranging from neural cell fate specification to proper matching between pre- and post-synaptic partners. How neuronal lineage and birth timing influence wiring specificity remains an open question. Recent findings suggest that the relationships between lineage, birth timing, and wiring specificity vary in different neuronal circuits. In this review, we summarize our current understanding of the cellular, molecular, and developmental mechanisms linking neuronal lineage and birth timing to wiring specificity in a few specific systems in *Drosophila* and mice, and review different methods employed to explore these mechanisms.

## Introduction

Multiple developmental processes, including cellular specification, axon and dendrite targeting, and synaptic partner matching, must be tightly coordinated to ensure precise neural circuit assembly. Accordingly, many studies have focused on exploring the developmental mechanisms underlying wiring specificity, revealing, over the past several decades, numerous molecular and cellular mechanisms that regulate neural cell fate specification, axon guidance, and dendrite morphogenesis [1–3]. Synaptic partner matching, the final step in circuit assembly, remains relatively poorly understood, and underlying molecules and mechanisms are just being revealed [4–7].

In this review, we discuss how neuronal lineage and birth timing are linked to wiring specificity at the cellular and molecular levels. Progenitors undergo a series of cell proliferation and differentiation events in the process of generating postmitotic neurons. Cell lineage denotes this series of events for an individual cell or cell type. Here, we use the term *lineage* to refer to the last few rounds of cell divisions that generate postmitotic neurons from a proximal progenitor. Many molecular factors and cellular mechanisms synergize to ensure that each step, from progenitor proliferation to wiring of immature neurons, is tightly controlled. In some neuronal systems, different neuronal subtypes are sequentially generated from one progenitor or a pool of common progenitors, and birth order or birth timing can predict their cell fates and wiring patterns; we classify such lineage-related processes, which specify neuronal cell fate and wiring, as *intrinsic* mechanisms. In other neuronal systems, cell fate and consequent wiring patterns have been demonstrated to independent on lineage. As processes such as lateral inhibition, extracellular induction and stochastic regulation have been shown to play important roles in wiring these circuits, we classify these as *extrinsic and stochastic* mechanisms. In this review, we discuss how intrinsic, extrinsic, and stochastic mechanisms contribute to wiring specificity within lineages in both *Drosophila* and mouse nervous systems, using findings from six relatively well-studied systems and dividing these findings into intrinsic and extrinsic/stochastic sections based on our current understanding. We note that various combinations of intrinsic, extrinsic and stochastic mechanisms may be used in most or all developing neuronal systems; our categorizations of a specific system as using intrinsic or extrinsic/stochastic mechanisms below reflect either the biased use of one mechanism over the other or that our understanding of one mechanism is more complete than our understanding of the other in that system.

## Intrinsic regulation of birth timing-dependent neural wiring

Some neuronal circuits appear to rely heavily on intrinsic mechanisms in the establishment of wiring specificity. Here we review how birth timing-related intrinsic factors guide development of wiring specificity in several model systems, including *Drosophila* olfactory projection neurons (PNs), mushroom body (MB) neurons and mouse cortical excitatory neurons. In reviewing findings from each system, we first describe the established relationships between cell lineage or birth timing and wiring specificity, and then summarize potential mechanisms at the molecular and cellular levels underlying such regulation.

### Drosophila olfactory projection neurons

In the *Drosophila* olfactory system, 50 classes of olfactory receptor neurons (ORNs) form one-to-one connections with 50 classes of second-order projection neurons (PNs) in the antennal lobe in 50 discrete glomeruli [8–10]. Each PN class restricts its dendrites to one glomerulus and features a stereotyped axonal arborization pattern in the lateral horn, a higher brain center that processes olfactory information [11–15]. *Drosophila* PNs have provided an excellent system for investigating the relationship between cell lineage and wiring specificity. Studies of this system have demonstrated that dendrite targeting of different classes of PNs can be completely predicted from their birth order or timing within the PN lineage [12, 16, 17].

**Fig. 1:**
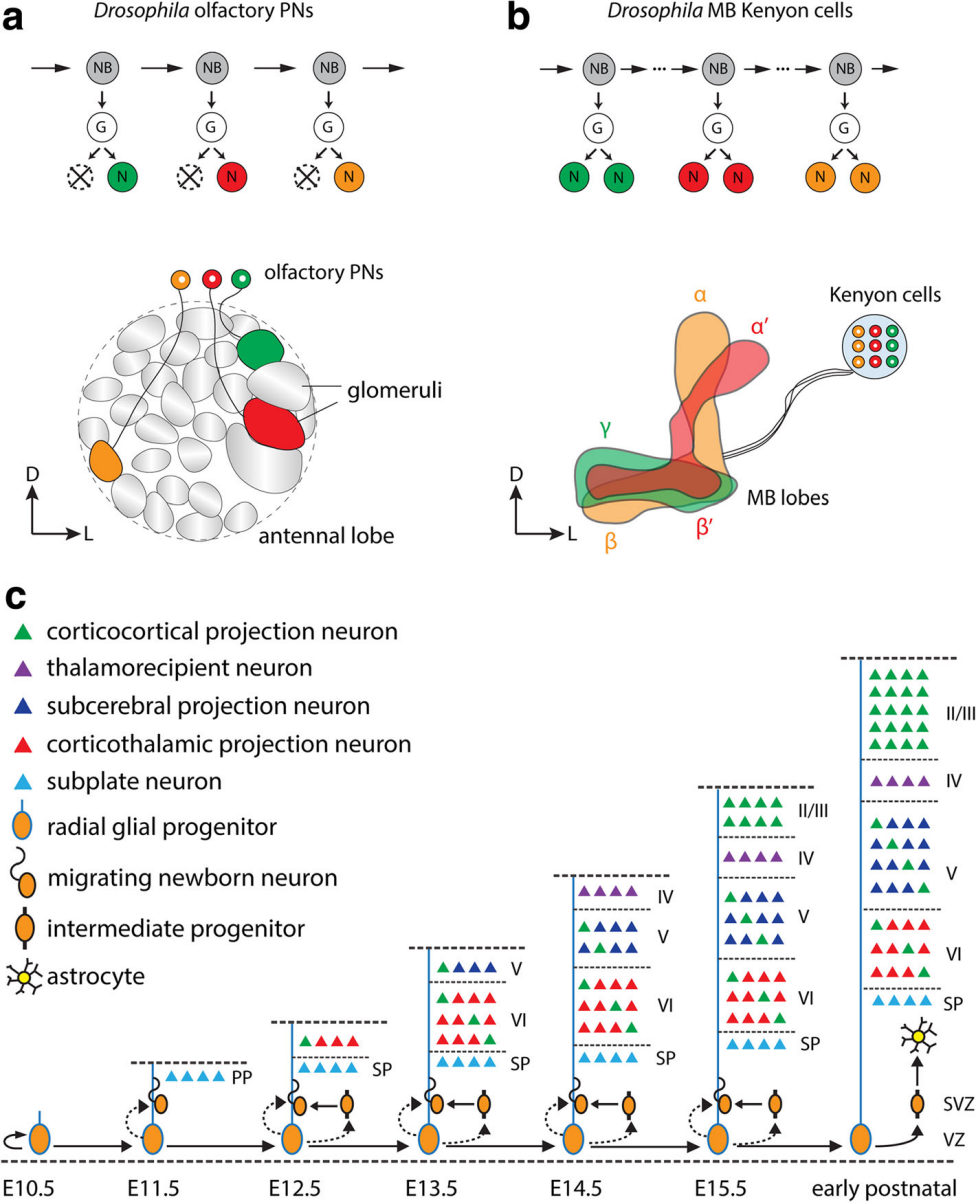
Intrinsic regulation of birth timing-dependent neural wiring. **a** and **b** In *Drosophila*, different types of olfactory projection neurons (PNs; **a**) and mushroom body (MB) Kenyon cells (KCs; **b**) are sequentially born from a common neuroblast (NB) in a stereotyped manner. In the anterodorsal PN (adPN) lineage, one of the postmitotic neurons undergoes apoptosis, so that only one PN is generated from one ganglion mother cell (GMC; labeled as G). Different PN classes send their dendrites to specific regions (glomeruli) in the antennal lobe. In the KC lineage, both postmitotic neurons resulting from GMC division survive and project their axons to the same MB lobe. D: dorsal; L: lateral. **c** In the developing mouse cortex, radial glia in the ventricular zone (VZ) divide asymmetrically to give rise to newborn projection neurons that populate progressively more superficial layers of the mature cortex and intermediate progenitors in the subventricular zone (SVZ), which themselves further divide to generate newborn projections neurons. Corticocortical projection neurons in layers II/III and scattered throughout layers V and VI project within the cortex; subcerebral projection neurons primarily occupying layer V project to subcortical structures such as the superior colliculus, pons and spinal cord; and corticothalamic projection neurons primarily occupying layer VI project to the thalamus. Radial glia produce astrocytes last, after filling in the cortex with projection neurons. Arrows represent postmitotic progeny; arrows with dotted lines represent possible postmitotic progeny. SP: subplate; PP: preplate; EX (e.g. E13.5): embryonic day X (days post conception, e.g. embryonic day 13.5) in mouse

Using mosaic analysis with a repressible cell marker (MARCM; see Box), Jefferis et al. found that PNs are derived from three separate neuroblast lineages, named the anterodorsal, lateral and ventral lineages according to their cell bodies’ positions relative to the antennal lobe [12]. Anterodorsal and lateral PNs (adPNs and lPNs) are excitatory neurons that send their dendrites to single, distinct glomeruli, whereas ventral PNs (vPNs) are inhibitory GABAergic neurons that send their dendrites to one or more glomeruli [13, 18]. Within each lineage, one neuroblast repeatedly undergoes asymmetric division, giving rise to a new neuroblast and a ganglion mother cell, which divides again to generate two neurons (Fig. 1a). In the adPN and vPN lineages, only one of the two post-mitotic neurons survives and develops into a PN, while in the lPN lineage, both post-mitotic neurons survive, developing into one PN and one local interneuron [17, 19].

Since MARCM allows temporal control over the induction of mCD8GFP-marked single cell clones [20], investigating the cell body position and target choice of single PNs induced at different times has allowed researchers to correlate PN classes with their lineage and birth order. Interestingly, within each lineage, different PN classes are born sequentially in a stereotyped order [12]. Two later studies using twin-spot MARCM, which allows labeling of sister clones from a common progenitor with two different fluorescent proteins [21], characterized the birth order of adPNs and lPNs more comprehensively. The authors captured every single neuron from one lineage based on birth order and identified several additional PN classes in both lineages not previously characterized [16, 17]. Meanwhile, twin-spot MARCM enabled the authors to deduce the number of cells in individual PN classes, revealing that each class comprises a stereotyped number of cells ranging from one to seven. Consistent with previous findings, both studies showed that lineage and birth order predict PN cell fate and dendrite targeting.

The stereotyped birth order of different PN classes suggests that there must be lineage-related intrinsic factors controlling cell fates of PNs and their dendritic targeting. What are these intrinsic factors? Transcription factors and cell-surface/secreted molecules are widely believed to be key factors regulating cell fate and wiring specificity, respectively. Accordingly, various transcription factors and cell-surface/secreted molecules have been shown to play crucial roles in regulating PN axon/dendrite targeting [5]. Recent findings suggest that transcription factors act within each lineage to specify different PN classes, and cell-surface/secreted molecules act downstream of transcription factors to directly execute the molecular processes underlying wiring specificity [22].

For example, Abnormal chemosensory jump 6 (Acj6) and Ventral veins lacking (Vvl, also called Drifter), two POU domain transcription factors, have been shown to be lineage-specific factors for adPNs and lPNs, respectively [23]. Acj6 and Vvl not only show lineage-specific expression patterns, but also are required for dendrite targeting of adPNs and lPNs, respectively. Loss of Acj6 in the adPNs or loss of Vvl in lPNs causes significant dendritic targeting defects. Misexpression of Acj6 in lPNs or Vvl in adPNs leads to aberrant targeting of PN dendrites to the glomeruli normally occupied by the other PN lineage. Acj6 also controls the axon terminal arborization of adPNs in the lateral horn, indicating that one transcription factor can affect wiring of both dendrites and axons in the same cell type. Additionally, Lim1, another POU domain transcription factor, is expressed in and required for dendrite targeting of vPNs but not for the other two lineages [24]. Since each lineage generates multiple PN classes, individual lineage factors are insufficient to specify different PN classes and corresponding axon/dendrite targeting. Indeed, additional lineage-specific transcription factors expressed in a subset of cells within a lineage, such as Islet and C15, have been identified [22, 24].

Different expression levels of the same transcription factor can also help to specify PN classes. For example, a temporal gradient of Chinmo, a BTB-Zinc finger transcription factor, governs assignment of neuronal identity in both PN and mushroom body lineages (see below) [25]. Loss of Chinmo leads to a transformation of early-born neuronal fates to late-born neuronal fates, and misexpression of Chinmo causes the opposite effects. Interestingly, a recent study shows that in addition to lineage-specific transcription factors, two RNA-binding proteins, IGF-II mRNA-binding protein (Imp) and Syncrip (Syp), could also act as intrinsic factors to specify PN identity [26]. Imp and Syp show opposing temporal gradients across the progression of both PN and mushroom body lineages (see below), and they promote early and late neuronal fates, respectively. Imp and Syp appear to govern temporal neuronal fates at least partially through Chinmo. Another recent study reveals that the transcription factor Seven-up (Svp) is critical for establishing Imp/Syp temporal gradients [27]. In summary, PNs of specific classes, which target their dendrites to specific glomeruli, are born in an invariant order, and this process seems to be controlled by a combination of transcription factors and RNA-binding proteins.

### Drosophila mushroom body Kenyon cells

Like *Drosophila* olfactory projection neurons (PNs), different types of *Drosophila* mushroom body (MB) intrinsic neurons, also known as Kenyon cells (KCs), are also born sequentially and in an invariant order (Fig. 1b), suggesting that lineage-related intrinsic factors also influence the progression of the MB lineage. The *Drosophila* MB is a higher order center for olfactory learning and memory and other brain functions such as sleep and courtship [28–32]. The MB contains four main parts: somata, calyx, peduncle and lobes. KC somata cluster in the dorsal posterior brain and send processes anteriorly, forming dendritic branches that comprise the calyx and then converge to form the peduncle. The axon bundle bifurcates at the anterior end of the peduncle to form dorsal (α and α’) and medial (β, β’ and γ) lobes (Fig. 1b). KCs are classified as γ, α’/β’ or α/β neurons, according to the lobes in which their axons terminate. All KCs originate from four neuroblasts in each hemisphere and each neuroblast generates an indistinguishable set of KCs. Clonal analysis using MARCM revealed that these three types of neurons are born sequentially from these common neuroblasts in a stereotyped order [33].

γ neurons are born first, before the midlarval stage; next, in the late larval stages, α’/β’ neurons are born; lastly, during the pupal stages, α/β neurons are born [33]. In the larval brain, both γ and α’/β’ neurons send axons to both dorsal and medial lobes. Whereas α’/β’ retain their bifurcated axon branches during metamorphosis, bifurcated axons of γ neurons degenerate in the early pupal stage and axon fragments are phagocytosed by the glial cells. γ neurons then extend axons only medially to form the adult γ lobe [33–36]. KC dendrites integrate inputs from projection neurons encoding olfactory, thermal, gustatory and visual stimuli [32, 37, 38], while MB output neurons elaborate segregated dendrites that form 15 distinct compartments in the MB lobes [32, 39]. In summary, three classes of KCs form connections with upstream and downstream partners, and current evidence suggests that lineage information fully predicts cell fate and wiring specificity.

Intrinsic factors such as Chinmo, Imp and Syp, which specify PN fates, also specify neuronal fates in the MB lineage [25, 26]. Interestingly, studies of the *Drosophila* embryonic ventral nerve cord suggest that sequential expression of another set of transcription factors (Hunchback/Hb, Kruppel/Kr, Pdm, and Castor/Cas) drives temporal cell fate specification [40]. These factors are transiently expressed in neuroblasts; inheritance by post-mitotic cells is what ultimately specifies cellular identities [40, 41]. Recent studies have also shown that optic lobe neuroblasts use a similar temporal patterning strategy featuring yet another set of molecules to control neural fate in the medulla [42, 43]. These findings suggest that different neuronal systems in the developing *Drosophila* central nervous system use analogous temporal patterning strategies that nevertheless employ different sets of molecules.

Several questions regarding the development of *Drosophila* PNs and KCs remain unaddressed. What other intrinsic factors and mechanisms control neuronal specification? How do multiple factors cooperate to specify different neuronal classes? How do intrinsic mechanisms ultimately control wiring specificity? One recent study that applied single-cell RNA-sequencing to *Drosophila* PNs shed light onto these questions, suggesting that combinations of transcription factors and cell-surface molecules may play a critical role in specifying different PN subtypes [22]. However, how these two sets of molecules interface remains unclear, and should be investigated in future studies.

### Mammalian cortical excitatory neurons

Intrinsic mechanisms also regulate birth timing-dependent neural wiring in the developing mammalian brain. The role birth timing plays in organizing mammalian neuronal wiring is perhaps nowhere more apparent than in the developing cerebral cortex [44–48], which throughout embryonic and postnatal development forms a structure featuring six layers of excitatory neurons that largely project to different extra-cortical targets (Fig. 1c). Asymmetric divisions of individual radial glia (RG), the primary neural progenitor cells in the developing cortex [49], generate newborn excitatory neurons that migrate out from the ventricular zone along radial glial fibers, resulting in the formation of cortical columns [50]. RG also generate intermediate progenitor cells that also eventually differentiate into neurons [51–53]. Because the cortex develops in an inside-out manner, such that earlier-born neurons populate the deeper layers and progressively later-born neurons populate progressively more superficial layers, much work has investigated the relationship between birth timing and eventual cell position in various cortical layers. Astrocytes are born last, after all cortical neurons are born. Importantly, projection neurons that occupy different layers project to different targets: corticocortical projection neurons of layers II/III, V and VI project to the contralateral cortex; layer IV thalamorecipient neurons receive input from the thalamus and broadcast output to other layers (primarily layer II) of proximal cortex; layer V subcerebral projection neurons project to subcortical targets such as the superior colliculus, pons and spinal cord; and layer VI corticothalamic projection neurons project to the thalamus [54, 55]. Thus, these basic layer-specific projection patterns exemplify the effects of birth timing on both cell fate and neural wiring of cortex excitatory neurons.

The mechanisms underlying layer-specific neuronal specification appear to rely heavily on intrinsic properties of progenitor cells, and ongoing work investigates whether these properties apply uniformly to all RG. Two extreme models posit that a) timing is the sole determinant of the potential of a given RG cell, or b) pre-specified, potential-restricted RG subtypes preferentially generate neuronal subtypes with specific projection patterns. The most parsimonious model proposes that all progenitors have equal potential, and thus that birth timing is the only factor influencing progenitor competence. Support for this model comes from early transplantation studies in which early-stage progenitors transplanted into late-stage cortex could produce all neuronal subtypes, but late-stage progenitors transplanted into early-stage cortex could produce only superficial-layer subtypes [56–59]. These studies indicated that the competence of a given RG becomes progressively limited across cortical development, although later transplantation studies indicated that both intrinsic and environmental cues control RG competence [60, 61]. Retroviral labeling studies, in which early viral injections resulted in labeling of neurons of all layers and later viral injections resulted in labeling of superficial layer neurons, corroborated these results [62–65]. Finally, various in vitro approaches have recapitulated birth timing-dependent layering of cortical developmental processes [61, 66–68]. Taken together, these studies suggest that neuronal birthdate is an important determinant of neuronal positioning in the cortex, and thus of wiring patterns, but do not address the possibility of differences in the relative abundance of pre-specified, potential-restricted progenitor cells.

An alternative model, which still incorporates intrinsic, birth timing-dependent mechanisms, would posit that potential-restricted progenitors preferentially generate different neuronal subtypes, such that some progenitors give birth to neurons that predominantly populate lower layers while others give birth to neurons that predominantly populate more superficial layers. Sparse expression of subtype-specific transcription factors such as Fezf2, which defines adult subcortical projection neurons [69–71], and Cux1/Cux2, which define adult callosal projection neurons, suggests that different progenitor subsets may be at least partially committed to generating different neuronal subtypes [72, 73]. Further investigations of this hypothesis used Cre/CreER transgenic mouse lines (see Box) to trace Cux2^+^ and Fezf2^+^ lineages in order to investigate the eventual positions of neurons derived from Cux2^+^ and Fezf2^+^ progenitors. These studies yielded contradictory results, with an initial study reporting a population of cortical progenitors that preferentially generates neurons populating more superficial layers [74] and a subsequent study from another group using similar approaches, including experiments utilizing some of the same mice on different genetic backgrounds, reporting contrasting findings [75]. Taken together, these results highlight the necessity of careful performance and interpretation of fate-mapping experiments using mouse genetic tools [76, 77]. An additional study utilizing MADM-based clonal labeling provided evidence that RG divide in a stereotyped manner consistent with a more parsimonious, strictly timing-dependent model of cortical neurogenesis [78], but results from such MADM-based studies can potentially suffer from biases due to the genomic positioning of MADM cassettes; some loci may be more susceptible to recombination in certain cell types than others. Thus, while positioning of excitatory cortical neurons seems to be largely predicted by birthdate, the degree to which production of various projection neuron subtypes is limited to pre-specified progenitors remains an area of active investigation.

Recent studies of excitatory cortical neurogenesis have focused on the functional consequences of lineage-dependent cell positioning. Sister excitatory neurons in ontogenetic radial clones labeled by in utero intraventricular injection of eGFP-expressing retroviruses, for example, are preferentially connected and have stronger connections in the second and third postnatal weeks than unrelated neurons [79]. Furthermore, gap junctions mediate transient electrical coupling between sister excitatory neurons and are required for development of these preferential connections and subsequent similarity of functional response properties between sister neurons [80, 81], as predicted by prescient dye-tracing studies [82–85]. Such functional similarities may be most prominent in neurons born very closely in time, and thus most closely related by lineage [86, 87], although other factors, such as the distance between clones and thus the degree to which they share a developmental microenvironment may also predict functional connectivity patterns. Determining the relative contributions of lineage and local environmental factors will be difficult. Finally, as multiple reports have noted that neurons with similar response properties tend to be preferentially connected [88–91], it may be that lineage and birth timing predict preferential connectivity established by gap junctions together with shared response properties driven by thalamocortical specificity and plasticity-mediated maturation of functional corticocortical connections in the immature cortex [92]. However, the molecular mechanisms underlying these processes, thought to be executed at the length scale of spines [91], remain poorly understood. Taken together, these findings indicate that birth timing biases excitatory cortical neuron positioning and wiring, and that lineage relationships can predict functional connectivity and response properties.

Cortical interneurons, however, develop from distinct lineages originating in the medial ganglionic eminence, caudal ganglionic eminence and preoptic area [93, 94]. While several groups have been actively investigating the possible lineage-dependence of inhibitory interneuron positioning using a combination of viral fluorescent labeling and barcoding [95–101], differing results and divergent interpretations of common datasets highlight the need for careful application of lineage tracing tools (see Box) and analytic and statistical definitions and procedures. Thus, the possible lineage-dependence of cortical interneuron positioning and wiring has been the subject of intense investigation; additionally, any possible birth timing-dependence of cortical interneuron positioning and wiring is not fully understood and also warrants further study [102, 103]. Finally, studies of the developing vertebrate retina have also provided valuable insight into the intrinsic mechanisms underlying birth timing-dependent regulation of cell fate and wiring specificity, which has been extensively reviewed [104].

## Extrinsic and stochastic regulation of neural wiring

In other neural systems, birth timing and cell lineage do not appear to tightly constrain wiring patterns, suggesting that extrinsic and/or stochastic mechanisms play a more dominant role in regulating wiring specificity in these systems. Here, we discuss how such mechanisms influence the wiring specificity of *Drosophila* photoreceptor cells and olfactory receptor neurons (ORNs) and mouse cerebellar granule cells (GCs).

### Drosophila photoreceptors

**Fig. 2:**
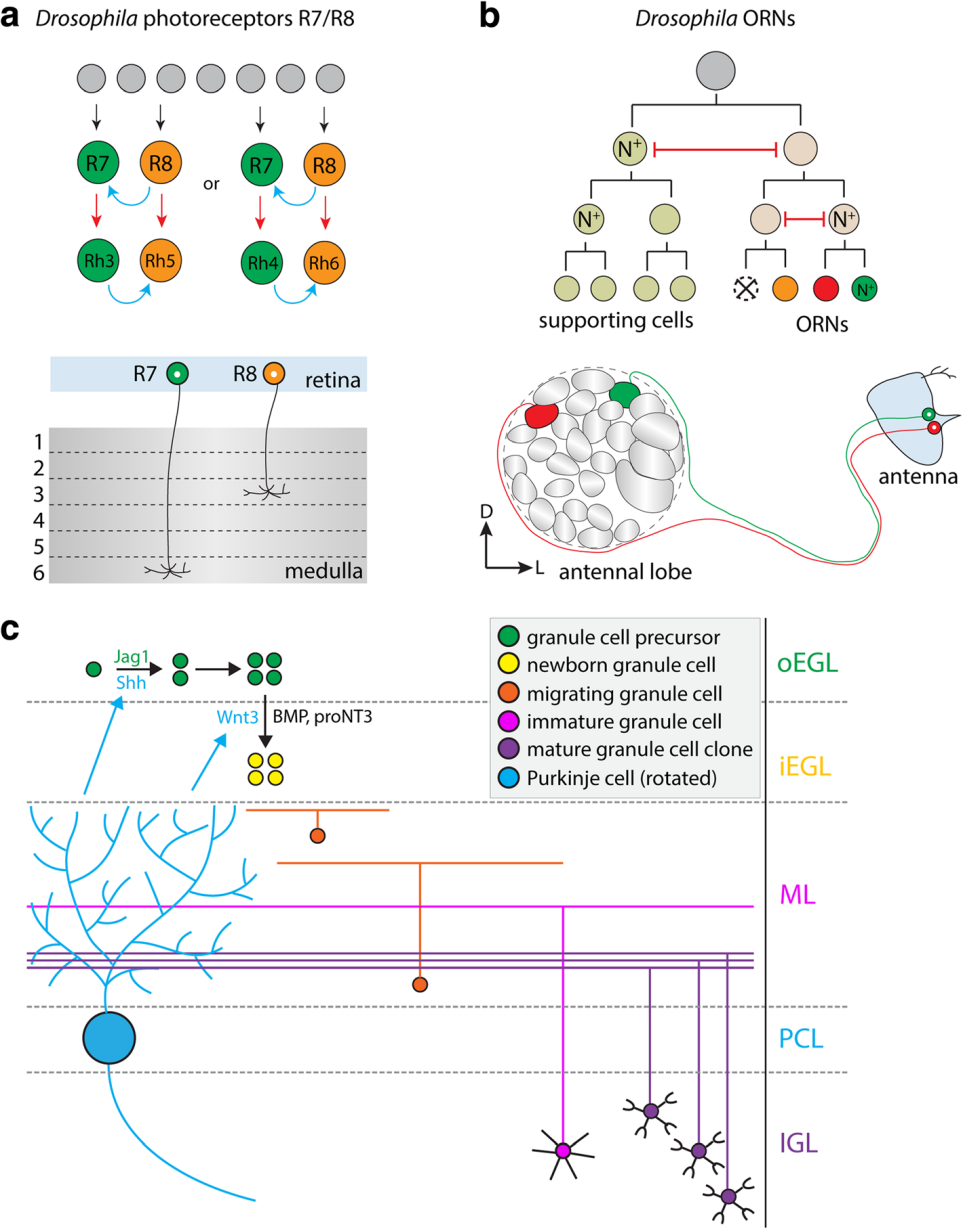
Extrinsic and stochastic regulation of neural wiring. **a** In the *Drosophila* retina, photoreceptors R7 and R8 (and R1-R6; not shown) are produced from a pool of progenitors. Cell-cell interactions (blue arrows) and stochastic mechanisms (red arrows) play critical roles in cell fate specification. Mature R7 and R8 cells project their axons to layers 6 and 3, respectively, of the medulla. Rhodopsin: Rh. **b** In the *Drosophila* olfactory receptor neuron (ORN) lineage, one progenitor cell within each sensillum undergoes several rounds of asymmetric division, giving rise to four non-neuronal supporting cells and between one and four ORNs depending on other events, such as cell death and glial fate adoption. Binary Notch signaling activation is iteratively employed, and lateral inhibition (red bars) is required for cell fate determination. Notch-ON (N^+^) and Notch-OFF ORNs send their axons to different glomeruli in the antennal lobe. D: dorsal; L: lateral. **c** In the developing mouse cerebellum, granule cell precursors (GCPs) in the outer external germinal layer (oEGL) undergo steady proliferation in a process promoted by Purkinje cell-derived Shh and GCP-derived Jag1. GCPs in the inner external germinal layer (iEGL) undergo a rapid burst of cell division before terminal differentiation, a process promoted by Wnt3 (expressed by Purkinje cells), BMP and proNT3. Migrating granule cells (GCs) then extend their parallel fiber axons into the molecular layer (ML), where they contact the dendritic arbors of developing Purkinje cells (rotated 90 degrees). Clones of mature GCs, which are born around the same time, project their parallel fiber axons to restricted depths of the ML. Parallel fibers of early-born GCs thus occupy the deepest depths of the ML while those of late-born GCs occupy the most superficial depths of the ML. PCL: Purkinje cell layer; IGL: internal granule layer

The *Drosophila* retina is a powerful model system for studying cell fate specification and wiring specificity. Current models suggest that cell fate specification of *Drosophila* photoreceptor cells involves a series of cell-cell interactions and some stochastic processes (Fig. 2a).

The *Drosophila* compound eye consists of around 800 identical units called ommatidia, and each ommatidium contains eight photoreceptors (R1-R8) arranged in a stereotyped pattern [105]. R1-R6 photoreceptors reside at the periphery of each ommatidium and project axons to the lamina, the first layer underneath the retina, where they form synaptic connections with lamina neurons. R7 and R8 photoreceptors reside in the center of the ommatidium and project their axons to the M6 and M3 layers of the medulla, the ganglion below the lamina, where they synapse with transmedullary neurons that send visual information to the lobula complex, a higher visual center. In developing ommatidia, the eight R neurons are generated in the following order: R8, R2/R5, R3/R4, R1/R6 and R7 [105, 106]. Interestingly, although eight classes of photoreceptors are produced in a fixed order, genetic mosaic analysis revealed that there is no lineage relationship between different classes [107]. These data suggest that inductive mechanisms, rather than cell lineage, specify *Drosophila* R cell fates. Below we review how cell-cell interactions and stochastic mechanisms specify R7 and R8 cell fates, as these cells have the best-characterized developmental mechanisms.

Two genes, *sevenless* and *bride of sevenless* (*boss*), are critical for R7 specification, as mutation of either leads to complete loss of R7 cells in all ommatidia [106, 108]. Mosaic analysis, allowing deletion of specific genes in one or several specific cells but not in neighboring cells, revealed more detailed mechanisms. Deletion of *sevenless* in non-R7 R cells does not affect the development of R7 cells, whereas deletion of *sevenless* in R7 cells always causes the transformation of R7 cells into non-neuronal cells, indicating that *sevenless* acts cell-autonomously. Conversely, *boss* acts cell-nonautonomously: its expression in R8 cells is indispensible for R7 development. Further molecular studies identified Boss as a 7-transmembrane ligand expressed in R8 cells, and Sevenless as a receptor tyrosine kinase expressed in R7 (and a few other cell types). Furthermore, the Ras/Raf/MAP kinase cascade acts downstream of the Sevenless receptor tyrosine kinase pathway that activates R7-specific genes [109, 110].

After R7 and R8 cells acquire their fates, cell type-specific rhodopsin (Rh) proteins are selectively expressed in those cells, allowing them to detect light of different wavelengths. Both R7 and R8 cells comprise two Rh-expressing subtypes: R7 cells can express Rh3 or Rh4 while R8 cells can express Rh5 or Rh6. These subtypes are paired precisely in ommatidia: 30% of ommatidia contain Rh3-expressing R7 paired with Rh5-expressing R8; 70% of ommatidia contain Rh4-expressing R7 paired with Rh6-expressing R8. Interestingly, the distribution of R7 subtypes seems to be regulated by the stochastic expression of transcription factor Spineless in R7 cells [111]. Spineless activates Rh4 and inhibits Rh3 expression in R7, and represses an unknown signal required to induce neighboring R8 cells to express Rh5. Conversely, Spineless-negative R7 cells express Rh3 and induce neighboring R8 cells to express Rh5. Consequently, Rh3-expressing R7 cells are always paired with Rh5-expressing R8 cells while Rh4-expressing R7 cells are always paired with Rh6-expressing R8 cells [111, 112].

As both inductive and stochastic mechanisms drive cell fate specification of *Drosophila* R7 and R8 cells, how, then, is cell fate specification linked to axon targeting? Several molecules have been shown to regulate R cell axon targeting, including trio, dock, Pak, insulin receptor (InR), Dscam, N-cadherin, Lar, Netrin/Frazzled, and Capricious [113–119]. While most of these factors have not been associated with cell specification mechanisms, Capricious provides an example of a molecule involved in both processes [120]. Capricious is a leucine-rich repeat transmembrane protein expressed in R8 cells but not in R7 cells. Gain- and loss-of function analyses suggest that Capricious regulates axon guidance in R8 cells. Strikingly, Capricious is activated by a transcription factor called Senseless, which is specifically expressed in R8 cells and acts as a key determinant for R8 cell fate by promoting R8-specifc rhodopsins and inhibiting R7-specific rhodopsins [120]. R7 cells express Prospero, another transcription factor, but downstream axon guidance molecules remain to be identified [120].

These findings suggest a model in which cell fate specification factors continually ensure that each cell type expresses a unique set of axon guidance molecules that drive wiring specificity. However, current studies largely focus on investigating either putative specification factors or ultimate wiring molecules. We expect that future studies integrating different techniques (see Box) will help to bridge investigation of both classes of molecules.

### Drosophila olfactory receptor neurons

Olfactory receptor neurons (ORNs) are the primary sensory neurons of the *Drosophila* olfactory system. There are 50 classes of *Drosophila* ORNs (~ 1300 cells) whose cell bodies are located in the antenna or maxillary palp. Each ORN class is defined by expression of a single olfactory receptor (*Or*) or a unique combination of ionotropic receptors and by the glomerulus to which their axons target in the antennal lobe [121–125]. Two fundamental questions regarding the development and wiring of *Drosophila* ORNs remain to be addressed: How are *Or* genes regulated in different ORN classes? And how is *Or* regulation coordinated with stereotyped axon targeting? One simple solution is to use olfactory receptors to instruct the axon targeting; indeed, this strategy appears to drive the development of the mouse olfactory system [126–129]. However, it appears that *Or* genes do not drive axon targeting in *Drosophila* [130]. Below, we discuss these two events separately and then speculate about how they may be linked.

ORN specification appears to utilize a combination of intrinsic, extrinsic and stochastic mechanisms and consists of multiple sequential steps: pre-patterning of the antennal imaginal disc by larval and pupal patterning factors including Hedgehog, Wingless, and Decapentaplegic [131]; sensillar assignment by transcription factors Lozenge, Atonal and Amos [132–134]; and final specification by additional mechanisms such as lateral inhibition via Notch signaling, epigenetic processes and additional transcription factors [135–137]. Sensilla are hair structures that cover the antenna and maxillary palp and host ORNs and supporting cells. Since different sensilla and their subtypes are distributed in a stereotyped manner on the antenna and maxillary palp and are associated with specific ORN types, sensillar specification is likely controlled by intrinsic factors. However, the further specification of ORN types within individual sensilla involves extrinsic factors. Here we discuss the final step of ORN specification, which leads to *Or* expression.

Within each sensillum, one multipotent precursor cell undergoes several rounds of asymmetric division, giving rise to between one and four fully differentiated ORNs and four supporting cells (Fig. 2b). Binary segregation of Notch activity (ON or OFF) is iteratively used during each round of division to regulate temporal and final cell fates [138], echoing a mechanism reported to drive development of the *Drosophila* peripheral somatosensory system [139]. During the initial division, the Notch-ON daughter cell acquires the supporting cell precursor fate and the Notch-OFF daughter cell acquires the neuronal precursor fate. The last round of division in the neuronal precursor lineage produces two distinct ORNs, one Notch-ON and the other Notch-OFF, expressing two different olfactory receptors and sending axons to different glomeruli in the antennal lobe. Genetic activation or inhibition of Notch activity leads to generation of two Notch-ON ORNs or two Notch-OFF ORNs, respectively. For example, mutation of the Notch positive effector *mastermind* leads to the generation of two Notch-OFF ORNs that project to the same glomerulus. Conversely, mutation of the Notch antagonist *numb* results in two Notch-ON ORNs that also project to the same glomerulus. Thus, Notch signaling is required for ORN fate specification, likely through lateral inhibition [138]. The exact number of ORNs within one sensillum varies and seems to be regulated by other mechanisms, such as cell death and glial fate adoption [140]. In summary, as different ORN classes are not born sequentially, birth timing and lineage do not predict ORN fate, as with PNs and KCs; instead, fate specification of ORNs born within a single sensillum through asymmetric division of a common precursor involves Notch signaling-mediated lateral inhibition [138].

Notch signaling occurs in all sensilla, but only assigns ORNs to two classes: Notch-ON and Notch-OFF. Thus, there must be additional context-dependent factors that complement Notch signaling, providing each precursor with the potential to acquire a different fate. One possibility is that the initial or intermediate precursor cell retains an intrinsic cellular memory that Notch signaling acts on during each cell division. Indeed, two recent studies showed that a cellular memory could be imprinted upon precursors through epigenetic regulation. One study discovered that the chromatin modifier Hamlet modulates cellular responses to Notch signaling in a context-dependent manner and controls *Or* expression choice. Hamlet executes locus-specific modifications of histone methylation and histone density to control the accessibility of DNA binding protein at the Notch target promoter regions [141]. Another study showed that the transcriptional corepressor Atrophin regulates *Or* genes in Notch-ON ORNs by controlling histone 3 acetylation [142]. Thus, these data suggest that regulation of chromatin and epigenetic status provides more diverse contexts for Notch signaling to act on, allowing specification of more ORN classes. We anticipate that a more comprehensive investigation of the chromatin statuses of ORNs and their precursors, for example, at the single cell level, would greatly enhance our understanding of the epigenetic regulation of these processes.

Transcription factors also play critical roles in regulating *Or* expression in post-mitotic ORNs, demonstrating that intrinsic and stochastic Notch-mediated mechanisms together guide ORN specification. Acj6 was first identified via an olfactory behavioral screen in which the *acj6* mutant displayed reduced jump responses to odor stimuli [143]. Acj6 is expressed in adult antenna and maxillary palp ORNs and is required for *Or* expression in a subset of ORN classes [144, 145]. Later work identified 13 alternative spliced isoforms of *acj6*, and overexpression of different isoforms in the *acj6* mutant background revealed that different isoforms specify different ORNs [146]. Individual isoforms could positively or negatively regulate the expression of certain *Or* genes. Pdm3, another POU domain transcription factor, showed broad expression in ORNs, but is specifically required for the activation of one *Or* gene, *Or42a* [147]. Interestingly, Acj6 is also required for *Or42a* expression, and *acj6* and *pdm3* appear to genetically interact. These data suggest that a combinatorial code of different transcription factors may regulate expression of *Or* genes. Accordingly, another study identified six new transcription factors that, in combination with Acj6, regulate *Or* expression in different ORNs [148].

How do transcription factors regulate *Or* expression in post-mitotic ORNs? If transcription factors directly regulate expression of specific olfactory receptors, there should be binding motifs in *Or* promoter regions. Three lines of evidence support this idea. First, an artificial *Or* promoter fused to a reporter could recapitulate expression of the endogenous *Or* even if the promoter-fused reporter was not inserted into the endogenous locus [149], suggesting that *cis*-regulatory elements in the *Or* promoter regulate *Or* expression. Second, several *Or* promoters have been shown to share a common binding motif which could be bound by an activator or a repressor depending on the positioning of the motif within the promoter [149]. Third, a specific set of *Or* genes have been shown to have an acj6 binding motif [150].

Taken together, these studies suggest that ORN cell fate specification involves interplay between intrinsic, extrinsic and stochastic factors. While we have discussed how distinct mechanisms drive ORN specification, it remains unclear how these mechanisms relate to ORN axon targeting at earlier developmental stages. So far, a number of signaling pathways and molecules, including Sema-2b/PlexB and Hh signaling and N-Cadherin, Dscam, Robo, Ten-a/Ten-m and Toll-6/Toll-7, has been shown to regulate ORN axon targeting [5, 6, 151–156]. However, most of these factors have not been shown to regulate ORN fate. Interestingly, Acj6, in addition to regulating expression of certain *Or* genes, also regulates axon targeting of some ORN classes [157]. The exact mechanism underlying such regulation of axon guidance remains unclear and is presumably independent of regulation of *Or* expression. Another study reported that Notch signaling in Notch-ON ORNs suppresses the expression of Sema2b, a key regulator of ORN axon trajectory choice [152]. Since trajectory choice is a critical step in the process of ORN wiring specificity, this finding linked ORN fate determination and wiring specificity.

Many interesting questions remain: What other transcription factors independently regulate *Or* genes? What is the combinatorial code regulating *Or* expression? Are there common upstream factors that regulate both *Or* expression and wiring specificity molecules? We anticipate that systematic analysis of single ORN transcriptomes during development will help to address these questions.

### Mammalian cerebellar granule cells

Inductive factors are well-documented to regulate differentiation, migration and wiring processes during development of the mammalian cerebellum. Like the cortex, the cerebellum is a layered structure with different cell types residing in different layers. Notably, cerebellar granule cells (GCs), small excitatory neurons packed into the internal granule cell layer, comprise over half of all neurons in mammalian brains. GCs send parallel fiber axons to the molecular layer, where they synapse onto dendritic spines studding the planar dendritic arbors of Purkinje cells, the inhibitory output projection neurons of the cerebellar cortex (Fig. 2c).

During prenatal cerebellar development, the rhombic lip generates granule cell progenitors (GCPs) that migrate to and undergo prolonged clonal expansion in the external germinal layer before exiting the cell cycle. GCPs then migrate through the developing molecular layer to form the internal granule layer, establish parallel fiber synapses with Purkinje cells and receive mossy fiber inputs via specialized dendritic claws (Fig. 2c; [158, 159]). As with cerebral cortical development, cerebellar cortical development proceeds in an “inside-out” fashion, as earlier-born GCs project their axons to deeper portions of the molecular layer and progressively later-born GCs project their axons to progressively more superficial depths [160–162]. GCP expansion seems to occur at a steady rate of around one or fewer divisions per day, followed by rapid expansion of clonally-related GCPs shortly before differentiation and migration [163].

Interestingly, single GCPs labeled at time points as early as E13.5 give rise to clones that project their axons to restricted depths of the molecular layer, indicating that these clones differentiate within a restricted time window (Fig. 2c; [164]). This finding suggests that clonally-related GCs may innervate nearby regions of a given Purkinje cell dendritic arbor [163], and while the functional significance of such lineage-related clonal axonal clustering remains unknown, one study reported spatially clustered patterns of parallel fiber activity during sensory processing that could facilitate generation of dendritic spikes, nonlinear postsynaptic calcium signaling and synaptic plasticity in Purkinje cells [165]. While the axons of GCs born around the same time project to restricted depths of the molecular layer, it remains unknown whether or not clonally- or birth timing-related GCs receive common mossy fiber inputs. To address this question, future studies should develop strategies to access early- and late-born granule cells and characterize their mossy fiber inputs.

Several secreted factors have been shown to regulate GCP differentiation, and thus to regulate the depth to which progeny GCs project their axons. One of the best-studied factors is Purkinje cell-derived sonic hedgehog (Shh), which serves to prolong GCP proliferation and inhibit GC differentiation [166–168]. Mutations in Shh and its downstream effectors have been observed in various forms of pediatric medulloblastoma, the most common pediatric brain tumor, which is caused by GCP over-proliferation. Shh signals via its canonical receptor Ptch1 and coreceptors Boc/Cdon and Gas1, which release Smo signaling in GCPs, leading to transcriptional activation via transcription factors Gli1 and Gli2 [169–172]. Additionally, in vitro studies revealed that GCP-derived Jag1 activates Notch2 signaling, which also supports proliferation [173].

Additionally, in vitro studies have identified secreted factors that promote GCP differentiation and migration. For example, BMP signaling inhibits GCP proliferation in vitro and induces differentiation by proteasome-mediated degradation of Math1, a transcription factor active in proliferating GCPs, and this signaling is disrupted in mouse models of medulloblastoma [174]. Wnt3, which is expressed in developing and adult Purkinje cells [175], also suppresses GCP proliferation and inhibits medulloblastoma growth, and does so by inhibiting transcriptional responsivity to both Shh and Math1 [176]. Interestingly, Wnt3 expression in Purkinje cells increases postnatally and is lost in mutants lacking GCs, implying that Wnt3 expression depends on interactions between GCs and Purkinje cells [175]. Finally, proNT3 promotes differentiation by inhibiting Shh-induced proliferation following activation of the pan-neurotrophin receptor p75 [177]. In vitro studies showed that proNT3 prevents Shh-induced proliferation of GCPs and upregulation of Shh pathways and genetic deletion of p75 in GCPs resulted in increased GCP proliferation [177]. However, the cellular source of the proNT3 required for this process remains unclear. Interestingly, GC-derived NT3 is also required for proper Purkinje cell dendritic morphogenesis [178], highlighting the multifunctionality of NT3 signaling in cerebellar development. Taken together, these studies reveal several secreted factors that promote GCP differentiation and migration yet primarily feature in vitro experiments, leaving the cellular sources of these factors indeterminate [179–182]. In the future, in vivo loss-of-function experiments utilizing cell-type specific Cre lines and floxed genes should be performed to recapitulate reported in vitro phenotypes.

Thus, various extracellular factors secreted by a variety of sources have been shown to regulate GC proliferation and differentiation, and thus also birth timing and axonal projection depth, as clonally-related GCs exit the cell cycle around the same time and thus also project their axons to restricted depths of the molecular layer. Specifically, these studies suggest that GCPs, unlike cortical progenitors, which divide asymmetrically, resulting in specification of postmitotic cell position and wiring based largely on birth timing (see transplantation studies described above), are highly sensitive to various local environmental cues secreted by Purkinje cells and GCPs themselves. Such cues either positively or negatively regulate GCP proliferation and differentiation, and future studies should focus on unambiguously identifying the cellular sources of these signals and the corresponding upstream mechanisms that in turn regulate activation of these signals.

## Box: Methods for lineage tracing in developing neural circuits

To address the role neuronal lineage plays in establishing wiring specificity in a developing neural circuit, neurons belonging to a specific lineage must be unambiguously marked at specific developmental stages, enabling subsequent characterization of neuronal morphology and wiring. Moreover, gene disruption in a targeted neuronal population allows researchers to address the molecular mechanisms underlying circuit assembly. Here, we review several powerful approaches for lineage tracing in developing neural circuits and discuss how these may be combined with emerging methods for characterizing circuit organization.

Pioneering techniques for neuronal lineage tracing include tissue transplantation and retroviral labeling [57, 183–187]. Prior to the development of genetic approaches, tissue transplantation allowed tracking of neural fates in developing nervous systems in situ. However, transplantation studies often require complicated, invasive embryonic surgical manipulations, limiting their resolution, flexibility, and applicability. Retroviral labeling strategies feature a retrovirus that infects a neuroblast and integrates its own genome into the host cell’s genome, resulting in inheritance of the viral payload by all progeny in the cell’s lineage. Recent approaches to retroviral labeling often utilize barcoded sequences as cell markers, expanding the throughput of viral lineage tracing and minimizing the likelihood of false clonal assignment. Consequently, retroviral labeling is still widely used for tracing neuronal lineage in developing mammalian neural systems.

**Fig. 3:**
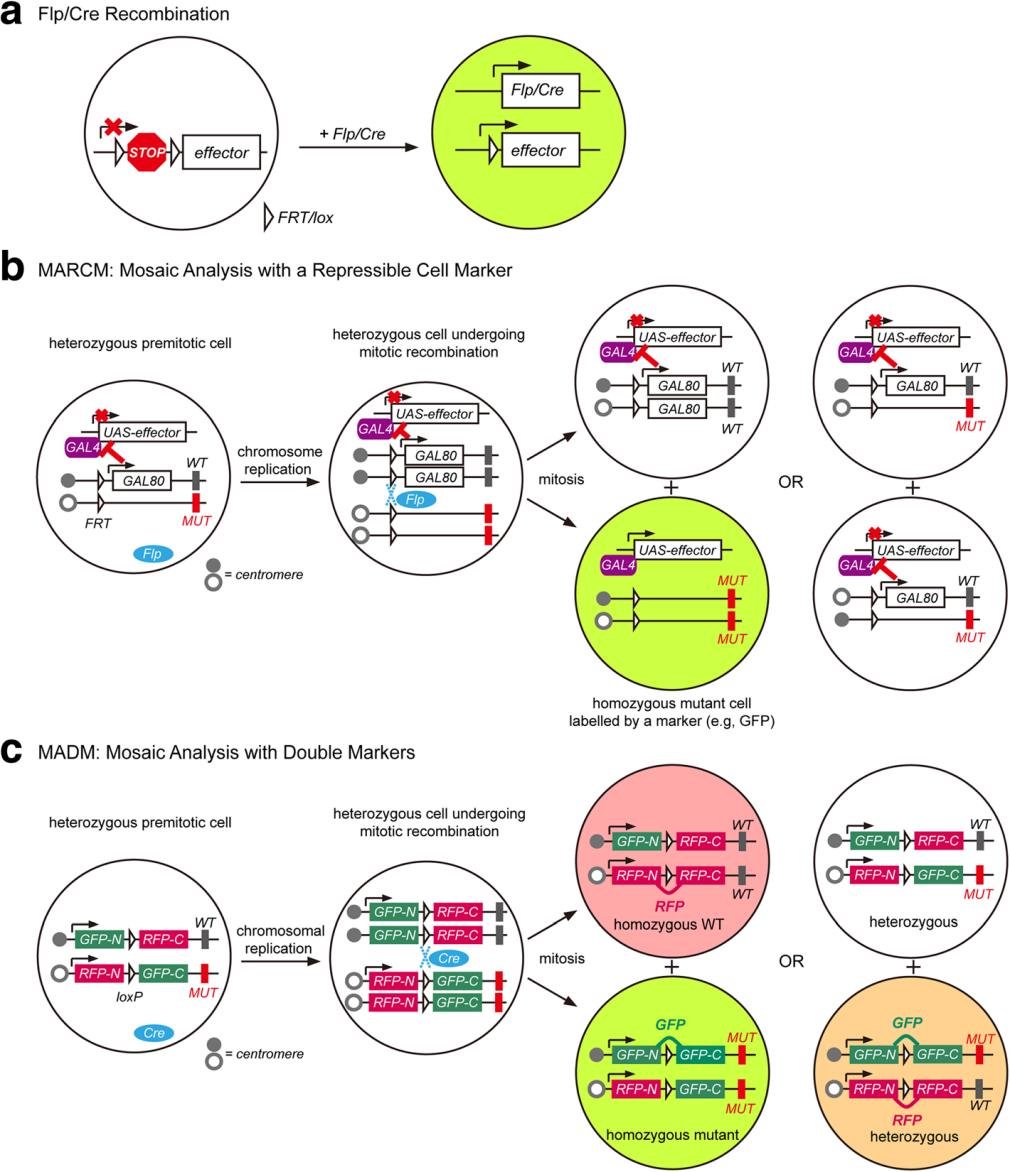
Genetic strategies for lineage analysis. **a** A transcriptional terminator (STOP) flanked by unidirectional FRT/lox sites blocks the expression of an effector/reporter gene such as GFP. In the cell population expressing Flp/Cre, the recombinase removes the terminator sequence to activate effector/reporter expression. **b** MARCM uses GAL80 to suppress marker expression driven by the GAL4-UAS binary expression system. The wild-type (WT), but not mutant (MUT), allele of the gene of interest is linked with GAL80. After Flp-mediated mitotic recombination, only the homozygous MUT progeny lose GAL80 and are labelled by marker gene expression. **c** In the original MADM configuration, N-terminal and C-terminal coding regions of GFP and RFP are segregated on homologous chromosomes. Cre-mediated mitotic recombination reconstitutes these coding regions to generate four distinct types of progeny (GFP^+^ only, RFP^+^ only, GFP^+^/RFP^+^ double-positive and unlabeled), in which fluorescent labeling corresponds to cellular genotype

Prototypical and subsequent genetic methods for clonal labeling have predominantly relied upon enzymatic DNA recombination by, most commonly, Flp and Cre recombinases. This recombination consists of removal of transcriptional terminator sequences flanked by unidirectional recognition target sequences (FRT and lox variants, respectively) or inversion of such sequences flanking an inverted reporter gene ORF, resulting in expression of reporter genes such as β-galactosidase (β-gal) or fluorescent proteins (Fig. 3a). DNA recombination is thus a simple and powerful genetic trick used widely in both invertebrate and vertebrate genetic model organisms for neuronal lineage tracing [188–196].

Many improvements have been made to basic recombinase-based strategies. For example, while many early genetic strategies relied on β-gal expression, which allows for sensitive, robust histological labeling of clones, β-gal localizes mostly to neuronal somata and does not robustly label axons and dendrites. Fusing the coding sequence of tau, a microtubule binding protein, to β-gal results in improved axonal labeling [197, 198]. Furthermore, fluorescent proteins such as GFP and tdT diffuse more easily into neuronal processes, and their membrane-tethered derivatives, such as mCD8-GFP and mtdT, diffuse profusely into neuronal processes due to the high surface area-to-volume ratios of these compartments [20, 189], allowing single-process resolution mapping of neuronal morphology. Recombinase activity can also be targeted to specific cell populations and developmental timepoints. For example, Flp/Cre expression driven by specific enhancers, promoters and genetic loci allows genetic access to targeted cell populations. Additionally, Flp expression driven by a heat shock promoter (hs-Flp) in *Drosophila* allows control over the time window and scale of clonal induction by heat shock at different time points and with varying durations. Analogous temporal control over Cre recombinase activity can be achieved using the estrogen receptor-fused Cre (CreER) and specifying injection times and agonist dosages [199]. Moreover, recombinase-based intersectional methods allow greater genetic specificity, thus enhancing the resolution of neuronal fate mapping [200–203]. Finally, recombinase-based clonal labeling strategies that combine mosaic genetic analysis and lineage tracing, such as mosaic analysis with a repressible cell marker (MARCM) in *Drosophila* [20] and mosaic analysis with double markers (MADM) in mice [164], are widely used to study developing neural circuits.

MARCM takes advantage of the yeast binary expression system GAL4/UAS, in which expression of GAL4 protein results in expression of a genetic element downstream of the upstream activator sequence (UAS), and the corresponding suppressor protein GAL80, as well as Flp/FRT-mediated inter-chromosomal mitotic recombination, to generate genetically distinct daughter cells/clones: homozygous mutant cells lack GAL80 while heterozygous and homozygous wild-type (WT) cells express GAL80. Thus, expression of the marker protein driven by UAS can be limited to the mutant homozygous lineage (Fig. 3b), allowing mosaic analysis of neuronal morphology and wiring [12, 20, 21, 23, 204]. Several MARCM variants exist, including reverse MARCM, in which most cells have a given gene disruption and only a few, labeled cells remain wildtype [205]; twin-spot MARCM, in which clones of interest and sister clones are visualized with complementary fluorescent markers [21]; and Q-MARCM, which uses the Q repressible binary system orthogonal to the GAL4/UAS system [206, 207]. MARCM has been used extensively for sparse and single-cell labeling for clonal analysis, as well as dissection of cell-autonomous and non-cell-autonomous gene functions. Since various *GAL4* and *Flp* driver lines can specify the cell-type and/or developmental stage MARCM targets, MARCM affords significant cell-type specificity and temporal resolution, and thus great flexibility for use in various *Drosophila* neural systems to study circuit assembly in WT conditions and to assess gene function in development, given the abundance of *GAL4* and *Flp* driver lines available to the *Drosophila* community.

MADM utilizes mitotic inter-chromosomal recombination for reconstitution of the coding regions of two distinct effector genes that are inherited by separate sister cells. These genes are typically fluorescent proteins that allow generation of a color code representing the genetic status of subsequent daughter cells or clones; in the original MADM6 configuration, for example, homozygous mutant cells are green, homozygous WT cells are red and heterozygous cells are either yellow or unlabeled (Fig. 3c) [164, 208]. Thus, this technique allows cell-autonomous analysis of gene disruptions in sparsely labeled cells expressing one of two fluorescent reporters (e.g. GFP and tdTomato). Since MADM requires two different gene cassettes to be inserted into homologous chromosomal loci near centromeres, it is limited to genes distal to these cassettes on chromosomes into which these cassettes have been integrated, with corresponding MADM mice generated. MADM-mediated clonal analysis is often accomplished using *CreER* driver lines and providing pulses of tamoxifen or its chemical analogs at specific developmental stages. This adaptation increases temporal control over MADM-mediated clonal labeling and genetic manipulations. Moreover, use of different *Cre* lines extends cell-type specificity to MADM. Finally, MADM alleles may also express effector genes, such as the tetracycline transactivator protein, instead of fluorescent markers, allowing, for example, simultaneous generation of a lineage misexpressing a gene of interest and a homozygous mutant sibling lineage [208]. MADM has been applied to study a variety of developing neural structures including the developing cortex, hippocampus, thalamus, cerebellum and enteric nervous system [78, 98, 163, 178, 208–219], as well as adult neural stem cells [220]. Finally, mice are being generated to allow MADM access to all autosomes (S. Hippenmeyer, personal communications).

Following labeling and genetic manipulation of a given lineage, assessment of neuronal wiring can take various forms. Fluorescent imaging and physiological recording are common and complementary ways to characterize neuronal wiring patterns. Live imaging can also be applied to monitor real-time dynamics of a labelled lineage [104, 221–224]. Multicolor stochastic labeling methods, such as Brainbow, dBrainbow and MCFO, allow analysis of neuronal network architecture on a large scale [225–229]. Recent innovations in light-sheet microscopy, tissue clearing techniques and image processing and registration enable performance of automated, high-throughput reconstruction in intact mouse brains [230–244]. These new technologies may allow detection and characterization of clones following extremely sparse clonal labeling and thus may eclipse traditional, more laborious methods in large volume tissue samples. Using a barcode-sequencing strategy, two recent studies achieved large-scale lineage mapping in vivo [245, 246], which could be coupled with emerging in situ RNA sequencing methods [247, 248] to enable brain-wide profiling of neuronal lineages and connections. In addition to anatomical analysis, in vivo functional imaging with genetically encoded calcium and voltage sensors has been widely used to study neuronal physiology [80, 249–255], offering additional means to address the functional association of sibling neurons, in additional to more traditional physiological approaches [79, 81]. Moreover, single-cell RNA sequencing has been applied to developing brains to identify molecular signatures of different types of neurons and their transcriptomic dynamics [22, 256–258], allowing systematic investigation of how neuronal lineage defines the molecular consortium controlling wiring specificity. Combining advanced genetic strategies with scalable profiling techniques provides an unprecedented opportunity to discover new principles of lineage-dependent neural circuit assembly.

## Summary and perspectives

Here we have discussed how neuronal lineage contributes to neural cell fate and wiring specificity in six different neuronal systems in *Drosophila* and mice. From birth to synaptic communication with appropriate upstream and downstream partners, a given neuron undergoes multiple steps to integrate into a functional neural circuit. Different neural systems have been observed to utilize different combinations of distinct intrinsic, extrinsic and stochastic mechanisms. Such a diversity of developmental mechanisms should be expected, given the diversity of information processing requirements these host neural systems attend to, and current investigations should both anticipate and appreciate discovery of new mechanisms that further enhance our understanding of these processes.

Understanding the mechanisms underlying neural cell fate specification and wiring specificity will be key to understanding how the brain develops and functions. While the diverse neural systems investigated have allowed discovery of a diversity of fate specification and wiring specificity mechanisms, undoubtedly many more remain undiscovered. Due to the complexity of the nervous system, most studies have focused on either how cell fate is specified within a lineage or how wiring patterns are established. Thus, future studies should aim to link these levels of analysis, and modern genetic tools combined with molecular profiling and anatomical characterization techniques should catalyze discovery of new mechanisms and principles underlying regulation of these developmental processes.

## Conclusion

Not applicable.

## Abbreviations

adPN: Anterodoral projection neuron
GC: Granule cell
GCP: Granule cell progenitor
KC: Kenyon cell
lPN: Lateral projection neuron
MADM: Mosaic analysis with double markers
MARCM: Mosaic analysis with a repressible cell marker
MB: Mushroom body
Or: Olfactory receptor
ORN: Olfactory receptor neuron
PN: Projection neuron
RG: Radial glia
vPN: Ventral projection neuron
